# An Overview on the Hallucinogenic Peyote and Its Alkaloid Mescaline: The Importance of Context, Ceremony and Culture

**DOI:** 10.3390/molecules28247942

**Published:** 2023-12-05

**Authors:** Marjolein Doesburg-van Kleffens, Amy M. Zimmermann-Klemd, Carsten Gründemann

**Affiliations:** Translational Complementary Medicine, Department of Pharmaceutical Sciences, University of Basel, Campus Rosental–Mattenstrasse 22, CH-4058 Basel, Switzerland; marjolein.doesburg-vankleffens@unibas.ch (M.D.-v.K.); carsten.gruendemann@unibas.ch (C.G.)

**Keywords:** *Lophophora williamsii*, peyote, mescaline, psychoactive, 5-HT_2A_ receptor, set and setting

## Abstract

Peyote (*Lophophora williamsii*) is a cactus that contains various biologically active alkaloids—such as pellotine, anhalonidine, hordenine and mescaline. Here, mescaline induces the psychoactive effects of peyote through the activation of the serotonin 5-HT_2A_ receptor and the subsequent release of calcium (Ca^2+^) from the endoplasmic reticulum (ER). Moreover, an evaluation of the therapeutic benefits of mescaline is also currently the subject of research. It is important to consider that the outcome of taking a psychedelic drug strongly depends on the mindset of the recipient and the context (set and setting principle), including ceremonies and culture. This overview serves to summarise the current state of the knowledge of the metabolism, mechanism of action and clinical application studies of peyote and mescaline. Furthermore, the benefits of the potential of peyote and mescaline are presented in a new light, setting an example for combining a form of treatment embedded in nature and ritually enriched with our current highly innovative Western medicine.

## 1. Introduction

**Figure 1:**
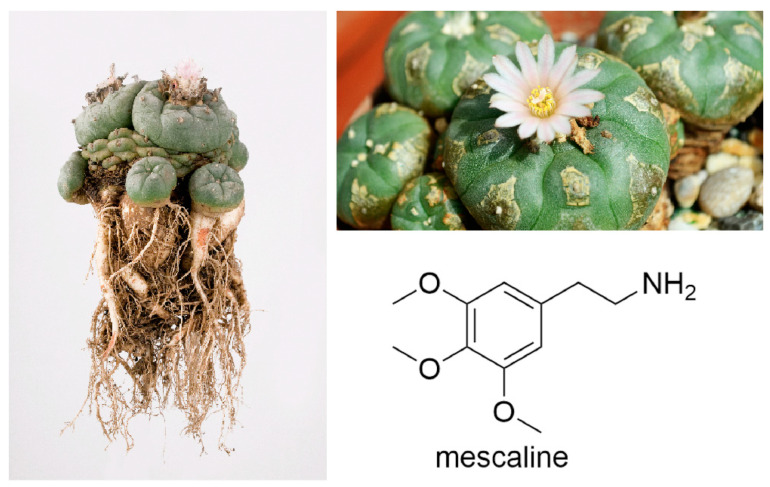
Pictures of the peyote cactus and the structure of its main alkaloid mescaline.

Peyote *(Lophophora williamsii)* is a cactus that contains psychoactive alkaloids—mainly mescaline (Figure 1). The ceremonial use of peyote was widespread at the time of Spanish rule in the Aztec Empire and in northern Mexico (around 1520) [1,2]. There are also descriptions of a wide range of medicinal uses, which include the treatment of burns, wounds, fever, rheumatism, snakebites and scorpion stings [3,4]. In the 1960s, the peyote cactus and its psychoactive substance mescaline made big waves. Its use was popular and much discussed in the media. In October 1964, the New York Times published the article “The ‘Diabolic Root”, which addresses the still very topical question of what is more dangerous: a weakly psychotropic cactus, substances such as tobacco and alcohol, or even narcotic institutions such as advertising and television (—today, the internet and social media would certainly be worth mentioning at this point) [5]. The use of peyote and mescaline was finally banned [6,7,8]. In 1978, the American Indian Religious Freedom Act authorised members of the Native American Church (NAC) to use peyote for religious purposes. Amendments to the American Indian Religious Freedom Act in 1994 permitted the harvesting, possession and consumption of peyote for “bona fide religious ceremonies” [9]. This was an important step by the Americans towards ending the war on drugs. A central role in this is played by the reform movement “Decriminalize Nature”, which has been very successful in decriminalising “herbal medicines” (e.g., psilocybin, ayahuasca and mescaline) [10]. In 1991, the publication of Dr. Alexander Shulgin and Ann Shulgin’s “summarised life work” book *PiHKAL* (acronym for phenethylamines I have known and loved): *A Chemical Love Story* [11] caused an uproar, as *PiHKAL* contains an auto bibliographical section as well as detailed instructions for the synthesis of 179 psychedelic phenethylamines (mostly discovered by Dr. Alexander Shulgin)—including mescaline and MDMA (“ecstasy”)—that are still commonly used today. For each substance, there is also information on the recommended effective dose and the subjective effects experienced by the authors.

An important question we all have to ask ourselves now, according to an article published in the New York Times, is what a so-called “drug peace” might look like. Is it possible to integrate psychoactive substances into our lives or even use them constructively [12]? Indigenous peoples often manage to integrate psychedelics into their lives as a sacrament, medicine or means of communication. Ritual consumption often seems to protect against drug problems [12]. What is important here is that consumption is not carried out casually and alone, but rarely and then mindfully and together. This approach could serve as a model for integrating psychedelics into everyday life [13].

However, it is particularly important to have a comprehensive knowledge of the mechanism of action, and the potentials and dangers of the substances.

The history of peyote research begins with botanical studies from the 1840s and the listing of peyote in the Mexican pharmacopoeia [14]. Subsequently, in 1887, John Raleigh Briggs published the first study on the effects of mescal buttons [14]. Since then, research into the constituents (e.g., the main alkaloids: mescaline, pellotine, anhalonidine and hordenine), the biological effects and the biosynthesis of the active alkaloids progressed rapidly. The German pharmacologist Louis Lewin (1850–1929) and the chemist and physician Arthur Heffter (1860–1925), as well as the Merck Laboratory in Darmstadt, Germany, provided the decisive insights into alkaloid isolation from peyote in 1897 [14]. Heffter was finally able to show that mescaline, with a half-life of 6 h, was exclusively responsible for the intoxication symptoms associated with peyote consumption [14]. In the 1990s, the mechanism of action of mescaline finally became known. As with other psychedelic agents, the effect of mescaline is based on the binding and activation of the serotonin 5-HT_2A_ receptor [15,16,17,18].

Subsequently, the release of calcium (Ca^2+^) from the endoplasmic reticulum (ER) is mainly induced, but other signalling pathways also seem to play a role (see Section 2) [19,20,21]. 

Research is also underway into the potential therapeutic value of mescaline. This step is consequential in light of the increasing acceptance of psychoactive drugs in general and their use for therapeutic purposes (cannabis can be named as a prime example here [22,23,24]). Moreover, in the Western world, we are confronted with an increasing number of chronic diseases [25], psychiatric problems [26] and drug addiction [27]. Today, it also becomes more and more evident that there is a link between mental and physical wellbeing. This is also recognised in other medical systems such as traditional Chinese medicine (TCM), Ayurveda and integrative medicine. Data from studies suggest that, on average, people suffering from mental illness have a diminished life expectancy of 10 to 15 years [28,29,30,31].

These facts clearly require the establishment of new concepts for dealing with health and illness, self-healing and psychedelic substances, which can strongly influence these processes. 

In this overview, we dive into the history and the effects of the peyote cactus and its major hallucinogenic alkaloid, mescaline. Although a single molecule, mescaline is the most active peyote substance; it seems that the whole plant, complemented with non-material factors, is needed for sustainable effects on mental health. We describe the importance of the concept of set and setting; specifically, the context and the ceremony. This could potentially enrich our Western culture, using the traditional application of peyote as an example, with its rich history [32]. In our eyes, this could be the inspiration for a new Western healthcare system.

## 2. Search Strategy

For this overview, we used a predefined search strategy and included studies published in the PubMed database and the clinicaltrial.gov trial registry (search keyword: Mescaline) up to October 2023. The following keywords were used as search terms with Boolean operators in the PubMed database: Mescaline[tiab] AND psycho-active [tiab], psycho[tiab] AND ritual[tiab], psycho-active[tiab] AND ceremony[tiab], Mescaline[tiab] AND clinic. Only sources and publications in German or English language were considered. Clearly irrelevant studies were eliminated by screening all the titles and abstracts of the publications identified through the database searches conducted. The remaining papers were assessed by reviewing their full-text versions. In addition, the reference lists of all eligible papers were manually reviewed to minimise the risk of overlooking relevant studies. Searches were conducted and data extracted by MDK and AMZK. The extracted data were reviewed by a third author (CG). Discrepancies were resolved through discussions and consensus. The results of the included publications were summarised in a narrative summary.

## 3. Mescaline—Metabolism, Clinical Reactions and Signalling

Peyote cactus is either eaten or drunk as an infusion (tea). An average intake of 3 to 6 of the cactus buds (approx. 10 to 20 g dry weight) contains a dose of mescaline equivalent to 200–400 mg mescaline sulphate [33] or 178–356 mg mescaline hydrochloride.

The physical responses to mescaline (mescaline hydrochloride: 2.5 mg/kg and 5 mg/kg) in 10 formerly morphine-dependent subjects were increased body temperature, higher systolic blood pressure and pupil diameter, and a lower knee pain threshold. Furthermore, this study described altered visual, temporal and sensory perception after the consumption of mescaline [34]. Another study with 18 volunteers found altered colour perception after the ingestion of 5 mg/kg mescaline [35]. Euphoria was frequently described, but anxiety and panic as well as depression rarely occurred [34]. In some cases, real visual hallucinations or pseudo hallucinations occurred [34]. The peak of the provoked effects was 2–2.5 h after ingestion of the drug [34]. At higher doses (over 400 mg), nausea and vomiting were reported as side effects [36,37]. In studies, lower right hemisphere performance was observed after the ingestion of 500 mg of mescaline-sulfate when the 12 healthy subjects solved a “face/no face” decision task [38,39,40]. An acute psychotomimetic state occurred 3.5 to 4 h after ingestion of the mescaline [40].

Mescaline (3,4,5-trimethoxy-beta-phenethylamine), responsible for the hallucinogenic effect, is absorbed relatively quickly [41] and distributed to the kidneys and liver, resulting in an increased half-life and a delayed onset of effects [42]. Due to the poor fat solubility of mescaline, transport across the blood–brain barrier is rather poor [33], but brain uptake appears to be relatively rapid according to studies with α-[14C]-mescaline in cats (25 mg/kg) [43]. The brain/plasma ratio one hour after intravenous injection was three and washout after 6 h appeared to be low, so that retention in the brain was long-lasting [43]. This was confirmed by studies with [3H]-mescaline in the brain of marmoset monkeys (*Callithrix jacchus*) [44]. Accumulation in the brain (hippocampus, amygdala, lateral geniculate and anterior cingulate cortex) could still be detected here 18 h after administration (8 mg, i.p.) [44]. It is assumed that the metabolism of mescaline differs organ specifically [45]. In a rabbit model, it was shown that an amine oxidase, which is expressed in the liver and also to a lesser extent in the lungs, plays a central role in the metabolism of mescaline [46]. The amine oxidase oxidatively deaminates mescaline to 3,4,5-trimethoxyphenylacetaldehyde [45]. This aldehyde is non-toxic, but also unstable, so that oxidation to 3,4,5-trimethoxyphenylacetic acid (TMPA) or reduction to the inactive 3,4,5-trimethoxyphenylethanol follows. It can be assumed that the metabolites contribute to the hallucinogenic effect of mescaline, as the peak of the mescaline effect does not correspond to the highest mescaline concentration in the brain. TMPA is demethylated to form the substance 3,4-dihydroxy-5-methoxyphenylacetic acid, which is finally linked to glutamine by the glutamine N-acyltransferase to be excreted as 3,4-dihydroxy-5-methoxyphenacetylglutamine [45]. In vivo studies on mouse livers and brains have shown that conversion of TMPA to 3,4,5-trimethoxybenzoic acid (3,4,5-TMBA) is also possible [47]. In the brain, detoxification of mescaline is assumed to occur mainly through N-acetylation [47]. Studies in mice and rats confirm the formation of N-acetylmescaline, its O-demethylated metabolites N-acetyl-3,5-dimethoxy-4-hydroxy-phenylethylamine and N-acetyl-3,4-dimethoxy-5-hydroxy-phenylethylamine [48]. The process of N-acetylation of mescaline has also been observed in humans [43]. The half-life of mescaline is on average 6 h and 87% is excreted within 24 h and 92% within 48 h [49,50]. In humans, an average of 26.2% of the administered mescaline dose was excreted as TMPA in the urine. However, this value is in all probability dependent on the route of administration (—oral intake with subsequent metabolisation in the liver leads to a higher deamination than with intravenous administration) [51]. In addition to TMPA, minor metabolites such as N-acetyl-3,4-dimethoxy-5-hydroxyphenylethylamine, 3,4,5-trimethoxybenzoic acid, 3,4-dimethoxy-5-hydroxyphenethylamine, and 3,4-dihydroxy-5-methoxyphenacetylglutamine were also detected in human urine [52].

The hallucinogenic effect of mescaline, the active ingredient of peyote, is mainly due to an agonistic effect at the serotonin 5-HT_2A_ receptor [53], to which it has a binding affinity in the low µM range (6.3 µM) [54]. The 5-HT_2_ receptors are G protein-coupled receptors (GPCRs) found in the cerebral cortex, *locus coeruleus*, basal ganglia, hippocampus, platelets and vascular smooth muscle [55]. Interestingly, in addition to hallucinogenic 5-HT_2A_ agonists, there are also 5-HT_2A_ agonists (lisuride, ergotamine) that do not produce hallucinogenic effects by activating the 5-HT_2A_ receptor [19]. The model of “biased agonism” provides an explanation for this observation [56,57]. It states that different agonists can stabilise different active conformational states. The subsequent signal transduction is thus dependent on the respective active conformational state. After activation by an agonist, 5-HT_2A_ binds preferentially to G_q/11_ proteins [19,20]. The subsequent activation of PLC triggers the formation of DAG and IP by the hydrolysis of PIP_2_, leading to the release of intracellular Ca^2+^ from the endoplasmic reticulum [19,21]. However, the efficiency of this signalling response by hallucinogens is low, which means that other signalling pathways must play a role. Studies have shown that phospholipase A_2_ (PLA_2_) [58], PLA_2_-mediated release of arachidonic acid (AA) [59] and the Gβγ-mediated activation of ERK1/2 as a function of G_i/o_ [58] also play a role in hallucinogenic effects. In addition, studies have shown that the PDZ-binding domain of the 5-HT_2A_ receptor interacts with the protein postsynaptic density 95 (PSD-95), thereby enhancing G_q/11_ binding [60]. Experiments with PDS-95 knockout mice have confirmed that, for instance, the induction of the *c-Fos* genes, and thus the hallucinogenic effect, is dependent on PSD-95 [61]. Downstream, hallucinogenic and non-hallucinogenic 5-HT_2A_ agonists activate different target genes [62]. In HEK293 cells and in the somatosensory cortex of mice, it was shown that treatment with hallucinogenic and non-hallucinogenic drugs leads to comparable gene expression of *c-Fos* and *IkBα*, but that the genes *Egr-1* and *Egr-2* are only induced by hallucinogenic drugs [19,62,63]. 

High doses of mescaline are known to increase the release and/or reuptake of serotonin, as evidenced by an increased concentration of 5-hydroxyindoleacetic acid (5-HIAA), the major metabolite of serotonin, in the brain [45]. Besides 5-HT_2A_, mescaline also binds to the serotonin receptor 5-HT_1A_, the α1-adrenergic receptor, the dopamine receptors D1/2/3 and the trace amine-associated receptor 1 (TAAR1), also all in a low µM range [54]. A low dopamine-releasing activity has also been described for mescaline [64]. The phenylethylamine moiety of mescaline forms the structural basis. However, there is no evidence of dependence on mescaline [45].

## 4. Mescaline—Chemistry and Synthesis

Mescaline is a ring-substituted amphetamine consisting of phenethylamine substituted at positions 3, 4 and 5 by methoxy groups [65]. It was initially assumed that a slight structural change in the mescaline would destroy the psychoactive effect [66]. In fact, however, a slight structural change leads to much more potent molecules [67]. In their book *PiHKAL*, Dr. Alexander Shulgin and Ann Shulgin describe a 2,5-dimethoxy substitution pattern with a small hydrophobic substituent on C-4 of the benzene ring as optimal in terms of a strong psychedelic effect [11]. Cyclisation of the side chain also proved to be advantageous in terms of potency and interaction with the 5-HT2_A_ receptor [68]. 

The biosynthesis of mescaline (and peyote alkaloids in general) in the peyote cactus is not yet fully understood. So far, candidate genes for tyrosine/DOPA decarboxylase, hydroxylases and O-methyltransferases have been discovered in peyote tissue [69]. 

Various methods have been published for the synthesis of mescaline in the laboratory. Most of the methods are based on the formation of the ethylamine side chain on the corresponding substituted aromatic systems [42]. The two-step reduction of 3,4,5-trimethoxybenzaldehyde yields mescaline [70]. Also, the two-step conversion of substituted hydrocinnamic acids into amides with subsequent Hofmann degradation can be used to synthesise mescaline [71]. Further ways of synthesising mescaline are the basic hydrolysis of benzoylmescaline [72] or synthesis from the bisulphite adduct of the aldehyde [73]. The latter requires substitution of the sulphite moiety, acetylation of the hydroxyl group and catalytic hydrogenation. Last but not least, the reduction of 3,4,5-trimethoxyphenylacetonitrile [74], 3,4,5-trimethoxy-β-nitrostyrene [75] or 3,4,5-trimethoxyacetamide [76] to mescaline has been described. More modern approaches lead to the preparation of the mescaline precursor 3,4,5-trimethoxyacetonitrile [77] or the acetophenone ketal ring opening of 2-methyl-2-(3,4,5-trimethoxyphenyl)-1,3-dioxolane [78].

## 5. Peyote Alkaloids

**Figure 2:**
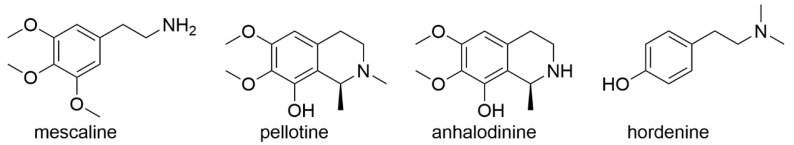
Structures of the main alkaloids of the peyote cactus.

Besides mescaline, several other alkaloids have been identified in the peyote cactus (Figure 2 and Table 1). Analogous to mescaline, the concentrations of these alkaloids can vary depending on the part of the plant. In addition, climatic conditions, light availability, soil conditions and harvest time influence the composition of a plant’s compounds [79]. Some of the alkaloids do not seem to have any pharmacological activity themselves but enhance the effects of mescaline [45].

The tyramine N,N-dimethyl derivative hordenine (Figure 2 and Table 1) is structurally similar to neurotransmitters such as dopamine and adrenaline and thus may lead to hypertension and increased respiratory and heart rates, as has already been shown in horses [80] and cats [81]. In addition, hordenine is of forensic interest. When barley grains are malting, hordenine is produced, which is subsequently also present in beer [81]. After beer consumption, hordenine can be detected in blood and urine and can therefore be used as a qualitative and quantitative marker for beer consumption [42,81]. Last but not least, antibiotic properties have been described for hordenine [82].

The second most common alkaloid of the peyote cactus is pellotine (Figure 2 and Table 1). The sleep-inducing effect of pellotine has been well studied. In one study, 49% of the subjects fell asleep 90 min after administration of pellotine (minimum dose 20 mg (s.c.), maximum dose 65 mg (s.c.)) and slept through the night. In 33% of these subjects, a dose of 20 mg (s.c.) was sufficient [83,84]. Another study confirms the sleep-promoting properties of pellotine, but mentions dizziness, nausea, vertigo and vomiting as minor side effects [85]. Further studies concluded that pellotine is also a valuable sleep aid for children [86]. The above-mentioned study results were promising, and pellotine was even marketed for a time by Boehringer und Sohn as a sleep aid [84]. However, the advent of cheaper synthetic barbiturates led to pellotine being taken off the market and not investigated further [87]. A new study that has just been published took up the investigation of pellotine as a sleep-promoting agent. The authors showed that pellotine concentration-dependently decreased locomotion in mice, inhibited REM sleep, and promoted sleep fragmentation [88].

Anhalinine is a mescaline analogue that is also found in the peyote cactus (Figure 2 and Table 1). Anhalinine likewise has stimulant effects by inhibiting cholinergic neuromuscular transmission [89].

**Table 1:** Alkaloid content of *Lophophora williamsii*, as percent total alkaloid (adapted from [84]).

	Alkaloid Content (Percent Total Alkaloid)
Mescaline	30.0
Pellotine	17.0
Anhalonidine	14.0
Hordenine	8.0

## 6. Clinical Studies and Therapeutic Potential of Mescaline

The investigation of the therapeutic potential of mescaline began in the 1950s with two studies on schizophrenic patients. After mescaline administration (mescaline sulphate intravenously: 500 and 500/750 mg, respectively), no therapeutic potential for schizophrenia was observed in either of the two studies [37,90]. In 1974, mescaline was then investigated in connection with alcoholism [91]. Participants in the study programme were given the opportunity to take part in NAC peyote meetings, alongside other activities. At these meetings, an average of 500 mg of mescaline was taken in the form of ground peyote powder or tea. Many of the participating American Indians from the Cheyenne and Arapaho Tribes stated that the peyote meetings were essential for overcoming their alcoholism. Increased openness and willingness to communicate were reported as well. A more recent study from 2005 also confirmed the positive effect of peyote on alcoholism [92]. Finally, a recent international study investigated the potential of mescaline in non-clinical settings for mental illnesses such as depression, anxiety, post-traumatic stress disorder (PTSD) and alcohol/drug use disorders [93,94]. Of the 452 participants, mescaline use positively affected 86% of people with depression (*n* = 184), 80% of people with anxiety (*n* = 167), 76% of people with PTSD (*n* = 55), 76% of people with alcohol abuse/disorders (*n* = 48) and 68% of people with substance abuse/disorders (*n* = 58). In the study register clinicaltrial.gov, six clinical studies (one, however, withdrawn) investigating mescaline are listed [95]. One completed study (ClinicalTrials.gov ID NCT0484901), with 16 healthy volunteers from Switzerland, investigated the subjective effects of different doses of mescaline using modern psychometric instruments, as well as the involvement of the 5-HT_2A_ receptor in the psychoactive effects of mescaline. Another reported double-blind, placebo-controlled, four-period crossover study with healthy volunteers from Switzerland (ClinicalTrials.gov ID NCT04227756) compared the acute effects of LSD, psilocybin, mescaline and a placebo. One study (ClinicalTrials.gov ID NCT05933213) will assess the effect of mescaline sodium enteric-coated tablets compared to morte-mescaline in the treatment of adult patients with lupus nephritis. The other three studies (ClinicalTrials.gov ID NCT02033707, ClinicalTrials.gov ID NCT05516823, ClinicalTrials.gov ID NCT05180149-withdrawn) will examine the influence of mescaline on the mood and performance of healthy volunteers, as well as the dependence of the use of the drug on the personality and personality perception of the user.

## 7. Ethnopharmacology

At the time of Spanish rule in the Empire of the Aztecs and Northern Mexico (around 1520), the ceremonial use of peyote was widespread [1,2]. In 1569, the Spanish missionary and ethnologist Bernardino de Sahagún described the use of peyote for religious and medicinal purposes by the indigenous peoples of Mexico in the Codex Florentinus [96]. During the Civil War (1860–1865), peyote was used by American Indians for both ceremonial and medicinal purposes. Medicinal uses were varied and included the treatment of burns, wounds, fever, rheumatism, snakebites and scorpion stings [3,4]. Kiowas used the plant to treat flu, scarlet fever, tuberculosis and venereal diseases. Indian tribes also used the plant to relieve the pain of childbirth, toothache and certain skin conditions [97]. Since the 1870s, the peyote cult has spread among the indigenous cultures of North America and is essential to the rituals of the Native American Church (NAC), founded in 1914 [98,99]. The plant is not conceived as a hallucinogen, but as a “teacher”; there is no distinction between medicine and religion, nor a separation between body and mind [100,101,102]. Thus, a special religious cult was created around peyote in the Native American Church (NAC) [102,103,104]. 

The use of peyote and mescaline was finally banned in the USA (Comprehensive Drug Abuse Prevention and Control Act) in 1970 [6], and in 1971 (Convention on Psychotropic Substances) [7] worldwide [8]. In 1978, the American Indian Religious Freedom Act, was passed to provide legal protection for the Church’s use of the plant. Since 1994, the American Indian Religious Freedom Act Amendments have allowed harvesting, possession and consumption of peyote as part of “bona fide religious ceremonies” [9]. The NAC, for whose members, the use of peyote as a sacrament is legal, has grown considerably in recent years, and it is estimated that they now have about 250,000 members in the US and Canada. The church is spreading rapidly, including among tribes where it was not before, such as the Navajo, among whom other religious movements are also on the rise, such as evangelical Christianity. Through the NAC, American indigenous cultural identity is kept alive. 

A ritual can be defined as an experience formally structured for the intention of healing or problem-solving [105]. For example, peyote rituals are used to help with alcohol and other addictions. The Navajo deploy the ritual as a powerful healing ceremony, which provides help with trauma and social disruption that is considered more effective than Western psychiatry [105,106,107].

Peyote ceremonies or rituals are substantially different from a standard psychedelic therapy session because of three important differences. There is little or no talking between participants. The (rhythmic) chanting is intended to alleviate anxiety that may arise from the hallucinatory effects of peyote and is considered an essential success factor in the healing or spiritual (divinatory) process. As a third distinction, rituals usually take place with little light or in the dark. The peyote ceremony differs slightly in this: to receive visions, one stares into the fire (at night) [105].

The peyote ceremony follows meticulous rules. Because sacred rites take place, it cannot be attended by just anyone. A detailed description can be found in Huttlinger and Tanner’s 1994 article, the peyote way [108]. In short, the ceremony is led by a “roadman”, who is assisted by a drummer, a firekeeper and a cedar-man. The latter is tasked with cleansing the space in a spiritual sense. The participants sit around a fire, in a tipi, and sing peyote songs while staff and rattle are passed around. This goes on all night from evening onwards and can last for about 14 h, during which no one is allowed to go outside, or sleep. The peyote is ingested one hour after the start of the ceremony in the form of powder or as tea. Among a specific group of indigenous Huichol Indians, this is augmented by story-telling, having first made an extensive pilgrimage to the original site of discovery in the so called sacred Wirikuta desert in northern Mexico.

**Figure 3:**
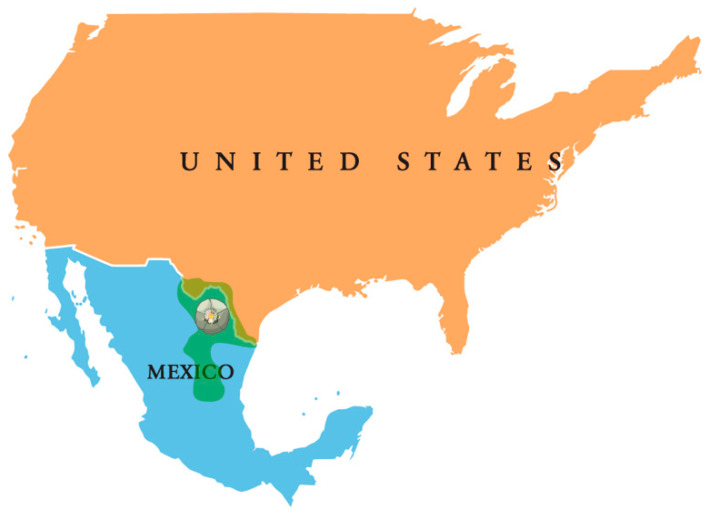
Geographic distribution of peyote (*Lophophora williamsii*) (highlighted in green).

Peyote grows very slowly and only in a limited number of places in southwest Texas and Mexico (Figure 3), where it grows in the desert and prefers limestone soil. Today, more peyote is used than ever before. Harvesting is increased and it is being sold in various countries (although, in certain countries, it is forbidden), thus leading to substantial diminished natural distribution and low-density populations of plants with also smaller size, creating vulnerability and ecological pressure [32]. 

## 8. Different Ideas about Disease—Chances and Challenges

To make sense of the indigenous peoples’ use of Peyote, we need to be aware that there are different concepts regarding health and disease that prevail and reign globally. The way Western medicine considers health and disease is reflected by the WHO definition of health (1948): “Health is a state of complete physical, mental and social well-being and not merely the absence of disease or infirmity” [109] and has undergone unprecedented development over the past two centuries. Whereas previously one’s body constitution (which is defined as “the inherent and relatively stable characteristics of individual human beings in morphological structure and function activities” [110]) was seen as the cause of susceptibility to disease, today, genetics, environmental factors and lifestyle are given a major role [111]. Several models exist, all based on the principle of cause (multifactorial or not) and effect (disease). Overall, we can state that today’s medicine still looks for explanatory causes [112]. In contrast to this current prevailing mechanistic understanding of medicine, many indigenous peoples and regenerative perspectives assume that health can only be created through a balanced equilibrium [102,108]. That is, our whole way of life and our relationships with our environment and fellow human beings contribute to such balances or imbalances [102]. So, the difference between indigenous and Western medicine is that the former considers all to be embedded in nature, connected with everything. Nowadays, we find this principle also in TCM, Ayurveda and integrative medicine. In Western medicine, this was also the case, until 250 years ago.

When assessing our physical health, we perform measurements and tests. However, concerning mental health problems, it takes courage on the part of both the doctor and the patient to talk about it, as there is still a stigma attached [30,113]. What makes it even more complicated is that the treatment is not just simple giving pill A for disease X and a pill B for disease Y, it usually consists of psychotherapy combined with medication. There is no guarantee that the prescribed treatment will actually work, unfortunately.

Prescribing tablets, however, is often the easier and less complicated approach, also reflected by the high usage of tranquillisers, sleeping pills, anti-depressants and alcohol, increasing every year. Worldwide, three million deaths every year result from the harmful and addictive use of alcohol. This represents 5.3% of all deaths [114]. Not to mention the opioid epidemic which plagues the United States as a serious public health concern [115].

The reason for the use of these kinds of drugs is very aptly described by Aldous Huxley in his 1954 book, *The Doors of Perception*, in which he describes his own experience of taking a small dose of mescaline [116]. Huxley claims that humans will never be able to refrain from chemical intoxicants because most people’s lives are perceived as painful and monotonous. This evokes a strong desire to escape suffering—even if only for a moment. This may sound rather melancholic and gloomy, but unfortunately it is probably the daily reality for a large part of humanity, which is evident by the above-mentioned high level of addiction worldwide. Another indication of the mental challenges and the extent of a sense of lostness that people experience is the fact that mental health disorders are the leading cause of morbidity and mortality [13,117].

In Western scientific medicine, the application of mind-altering substances such as the classic hallucinogens is relatively new. Their application is also difficult to reconcile with developments here, which are biomedically and pharmaceutically oriented [13]. One of the aspects that plays a role in this is the so-called set and setting principle: it turns out that the result of the administration of any psychedelic drug is not only determined by the drug, but rather very much depends on the intentions, expectations and general psychological state of being (state) of the receiver (the set). Also crucial is the immediate environment in which the drug is taken (the setting) [118,119,120,121,122,123]. The indigenous approach assumes that only one’s own body carries the potential to regenerate and heal its individual ecosystem. The doctor, healer or therapist can provide a “space” in which healing is possible; the setting, so to speak, that enables healing, when the so-called “set”, the factors related to the person that wants to be healed, is also right. In the traditional indigenous approach, however, strengthening one’s own healing potential in the harsh living conditions was largely culturally anchored and integral.

It is important to realise that the way of healing using the peyote cactus is also part of a specific worldview, mindset and lifestyle. It is therefore important that we learn to value this ancient traditional view and knowledge and research it further, respectfully and collaboratively [105,124]. This aspect is also addressed by Berlowitz et al. [125], who also advocate this multidisciplinary form of research and argue for bridging knowledge between traditional and Western medicine.

A question that arises is what the difference is between the (lasting) effect of ingesting peyote during a ceremony, and that of ingesting only the active component, the mescaline. In fact, this is the difference between the joint—probably synergistic—effect of the alkaloid mixture contained in the peyote cactus, completed with the so-called set and setting, compared to the effect of “just” the pure active substance. It is unimportant here, whether or not there is a placebo effect, possibly triggered by the presence of the roadman and his helpers, and the whole peyote ceremony [122]. This is what could enrich Western medicine, by creating a culture-specific setting, where healing can take place on multiple levels, making use of rituals more often. The “Western way of rituals” may involve the expertise and guidance of a doctor, in a safe, special, medical environment with good mutual agreements and proper personal preparation, for specific problems. 

Here, we took the use of peyote as an example. The risk of addiction to, or dependency on, mescaline usage is nihil, and intoxication produces mild symptoms that are not life-threatening [45]. This does not mean that we advocate the use of peyote, mescaline or other (semi-) natural substances like psilocybin or LSD, or synthetic equivalents with comparable effects, for psychiatric application.

More essential, in our opinion, is that by looking at how various cultures deal with illness, health and medicine, we can gain a broader overview and understanding of how disease can be treated. Ideally, we would like to combine original, proven indigenous knowledge and wisdom with our modern and Western approach and find ways to create the “best of both worlds”. An important aspect in all this is the reciprocity principle. Where we in the West could gratefully make use of indigenous wisdom in terms of “set and setting”, we could, for instance, share our knowledge in terms of quality and safety (including technique) with them, so that it does not become a one-sided “take”, but a shared opportunity for further development, which can benefit all humanity.

## 9. Future Research Aspects

With this overview, we hoped to present the research from a different perspective in order to offer the possibility of a change in consciousness and thus new chances and paths. How this change in consciousness might look was discussed using the example of peyote and mescaline. The mindset of indigenous people, which includes a connection with the natural world and a strong reliance on one’s own body as a “measuring instrument”, serves as an orientation here. The setting, which includes rituals and ceremonies, is also essential for the healing effect of the substances used.

A new approach to treatment could be a combination of outer, scientific evidence and internal evidence provided by the body, so that a patient is able, for example, to “feel” if a specific medicine prescribed for a given disease is right or not. There are ways to concretise such internal evidence. Here, it is important to understand that every plant has a potential to influence our body and mind. That is, if you taste peyote or just a “regular European plant” like, for instance, dandelion, a herb like thyme or rosemary, or mistletoe (a special plant used in anthroposophical medicine), you can describe what it does to your body; if you feel warm or cold, light or dark, fresh or exhausted, or other sensory effects. This can be achieved, for example, with the help of validated (sensory, psychological) questionnaires. The knowledge of the effects of plants gained in this way can then be used in the following for health promotion.

## 10. Conclusions

In short, for the molecular aspects of mescaline, the way it exerts its action in the cells, the signalling pathways that are involved and the effects it elicits in the human body, much has been published since the first description of its use in 1569. Its hallucinogenic effects are mainly due to its binding to the 5-HT_2A_ serotonin receptor. 

The highest proportion of mescaline is found in the peyote cactus (*Lophophora williamsii*), which in dried form contains 3–6% of the alkaloid mescaline. The ingestion of this cactus is part of a special (healing) ritual of indigenous peoples in specific parts of Mexico and Northern America. Peyote is classified as a hard drug and its use and possession is illegal (except for the members of the NAC). However, in the last two decades, the interest in the medical use of psychedelics has risen, along with the increasing numbers of complex health challenges we face, as humanity. 

In this overview, we looked at the application and the hallucinogenic effects of peyote and its major alkaloid mescaline. Although mescaline and its analogues have advantages (such as more precise dosing and possibly faster hallucinogenic effects), they lack the more sustainable effects of what is reported during peyote rituals. Thus, we think that peyote and its ceremonies serve as an example for a nature-embedded and ritual-enriched way of providing a possible treatment for mental health challenges. Thus, we could search for ways to combine this ancient knowledge with our present highly innovative Western medicine, to be able to develop a more sustainable healthcare system that can be of benefit globally.
